# Estimating shear modulus of isotropic materials from scanning laser Doppler vibrometry via convolutional neural networks

**DOI:** 10.1016/j.jmbbm.2025.107079

**Published:** 2025-06-04

**Authors:** Silvia Leccabue, Sara Moccia, Thomas J. Royston, Enrico G. Caiani

**Affiliations:** a University of Illinois Chicago, 1200 West Harrison Street, Chicago, 60607, IL, USA; b Politecnico di Milano, Piazza Leonardo da Vinci 32, Milan, 20133, Italy; c Scuola Superiore Sant’Anna, Via G. Moruzzi 1, Pisa, 56124, Italy; d Istituto Auxologico Italiano IRCCS, Ospedale San Luca, Piazzale Brescia 20, Milan, 20149, Italy

**Keywords:** Scanning Laser Doppler Vibrometry, Convolutional neural networks, Shear modulus

## Abstract

This study explores the use of Scanning Laser Doppler Vibrometry (SLDV) and Convolutional Neural Networks (CNNs) to estimate the stiffness of silicon-based materials. The research is motivated by the growing evidence that tissue mechanical property values are important parameters for diagnosis as they are sensitive to pathological changes. SLDV is a dynamic elastography technique that measures wave propagation and is non-contact, non-invasive, and relatively low-cost. CNNs have been shown to be able to assess mechanical properties from elastography images more accurately than traditional inversion techniques. Soft tissue-mimicking materials were used in the analysis to realistically simulate the properties of soft tissues, exhibiting similar deformation responses and stiffness values. Two different methods of mechanical vibration source were used to stimulate the specimens during imaging. The classification of the shear modulus of the materials was performed on two separate tasks: binary classification and a five-class classification. Open datasets of SLDV images were not present in accessible databases, so the proposed CNN architecture was pre-trained using synthetic wave data generated using a computational model and then fine-tuned with physical data. During the two experiments using physical data, the binary classification achieved an accuracy of 84.4%, and the multi-class classification reported an accuracy of 76.6%. While these results do not yet allow a clinical application for the estimation of the stiffness of organs and soft tissues, they constitute a step forward towards the implementation of an automatic and reliable method for assessing mechanical properties from elastography images.

## 1. Introduction

Noninvasively measuring and assessing changes in tissue transverse (e.g. shear) wave motion can potentially play a crucial role in monitoring the progression, and response to therapy or treatment, of many diseases and injuries that afflict virtually every soft tissue type and region in the body. Traditionally, the mechanical characterization of biological tissues is based on the response to tissue deformation by applying manual palpation (Hirsch et al., 2017). However, manual palpation does not allow clinicians to evaluate tissues and organs deep inside the body and represents a qualitative assessment (Dresner et al., 2001).

Dynamic elastography is an imaging method capable of quantitatively estimating and mapping tissue mechanical property values to use as a biomarker (Mariappan et al., 2010). Images are based on wave motion generated by a vibrating source (Garra, 2015). This measurement method has been implemented using ultrasound, magnetic resonance, and optical imaging modalities. Scanning laser Doppler Vibrometry (SLDV) is an optical approach to measuring motion on a surface with high resolution, accuracy and sensitivity over a wide bandwidth, from near static to MHz. SLDV has the capability to image vibratory motion below cardiac frequencies up through medical ultrasound frequencies (Tabatabai et al., 2013). Furthermore, this technique allows taking measurements without touching the surface, avoiding damage or interference with the tissue of interest (Hasheminejad et al., 2018). The Doppler effect and interferometry are the basic principles by which this approach can measure the amplitude and phase of the surface motion at a user-defined array of points. In this way, SLDV produces a spatial profile of the surface wave in terms of complex-valued displacements. This wave profile can be fit into a frequency response function to reconstruct material properties, such as a complex-valued shear modulus (Kearney et al., 2015), with the real and imaginary parts being the shear storage and loss moduli, respectively. Biological tissue is dispersive due to viscous losses and a change in the frequency means a change in shear storage and loss moduli (Tabatabai et al., 2013). Both wavelength and attenuation rate of a propagating mechanical wave are affected by the tissue elasticity and viscosity, which together result in a frequency-dependent “dynamic stiffness” (Guidetti, 2017).

Researchers have recently begun using Deep Learning (DL) techniques to process images acquired with dynamic elastography methods in order to reconstruct (i.e. solve the inverse problem) and map the mechanical properties of materials (Abueidda et al., 2019). Much of the excitement stems from the apparent ability of neural networks to make decisions and draw conclusions when processing complex, noisy, and even partial information (Eberhart, 2014). The ability to provide stable estimations and be less affected by a reduced Signal-to-Noise Ratio (SNR) during the acquisition process could lead to improvements compared to inversion techniques that have been used previously. Other limitations besides SNR that affect different inversion algorithms to varying degrees include the assumption of local homogeneity, the need for an initial guess of estimated properties, the need for spatial filtering, boundary effects and wave mode conversion. DL techniques potentially improve performance in resolution and accuracy by being less affected by poor SNR and these other factors (Murphy et al., 2018).

### 1.1. Main contributions

Convolutional Neural Networks (CNNs) have been widely used for medical image analysis, and a study has shown that CNNs can be adapted to leverage the intrinsic structure of medical images (Litjens et al., 2017). In the present study we aim to reliably estimate the complex shear modulus from physical data acquired through SLDV using CNNs. As a way to cope with limited availability of physical data, a different approach from training the model using only physical data was taken. Specifically, the contributions of this work were:

- Generating simulated data to account for the lack of physical data. The simulated data has to mimic the wavefield traveling within the material during the SLDV image acquisition process.
- Pretraining the CNN using synthetic and fine-tuning physical data acquired through the SLDV to distinguish the mechanical properties of different soft-tissue phantoms.
- Using the trained CNN classification-based model to estimate mechanical properties in terms of quantitative differences between five different silicon-based materials. This process overcomes one of the regression-based model limitations, i.e., to generate large outliers, and is minimally affected by the noise during the acquisition process, a crucial limitation of the traditionally utilized inversion techniques.

### 1.2. Deep-learning approach for the analysis of magnetic resonance elastography (MRE) and ultrasound elastography images

Murphy et al. (2018) proposed the use of an Artificial Neural Network (ANN) to estimate stiffness from MRE images by applying a patch size of roughly 1 cm in each dimension. Neural Network Inversion (NNI) was tested in simulation experiments and in vivo, comparing the results obtained with NNI and a traditional inversion method using a Direct Inversion algorithm (DI). The results showeed that NNI performed as well or better than DI in predicting the known stiffness of the data across all the expected four simulation experiments. Moreover, the results of the NNI provided a strong correlation with DI results in the liver, where the regression analysis demonstrated a strict relationship between the two methods of estimation. However, the designed model also produced large outliers in terms of estimated shear modulus.

Another recent research study based on DL tools applied to the problem of estimating MR elastography parameters was proposed by Hou et al. (2022). The authors used chicken breast muscle tissue as the material of interest to account for an intrinsic property of striated muscle: anisotropy. The parameters estimated from elastography images were shear modulus *μ*, shear anisotropy *ϕ*, and tensile anisotropy *ξ*. The obtained results were promising and demonstrated the reliability of DL on geometrically complex ex vivo measurements. However, like most studies using traditional reconstruction methods, the estimated parameter values were valid for static deformation of the muscle at only one position.

Vasconcelos et al. (2021) estimated the elasticity and viscosity parameters from simulated ultrasound shear wave elastography through a CNN for elasticity regression, and a CNN for viscosity regression. CNN models are robust to noise, vertical position, and partially to focal length; however, due to ultrasound detection and physiological motion, the noise affected the estimation, therefore the standardization of images and simulation models still needs some improvements.

## 2. Materials and methods

**Fig. 1.**
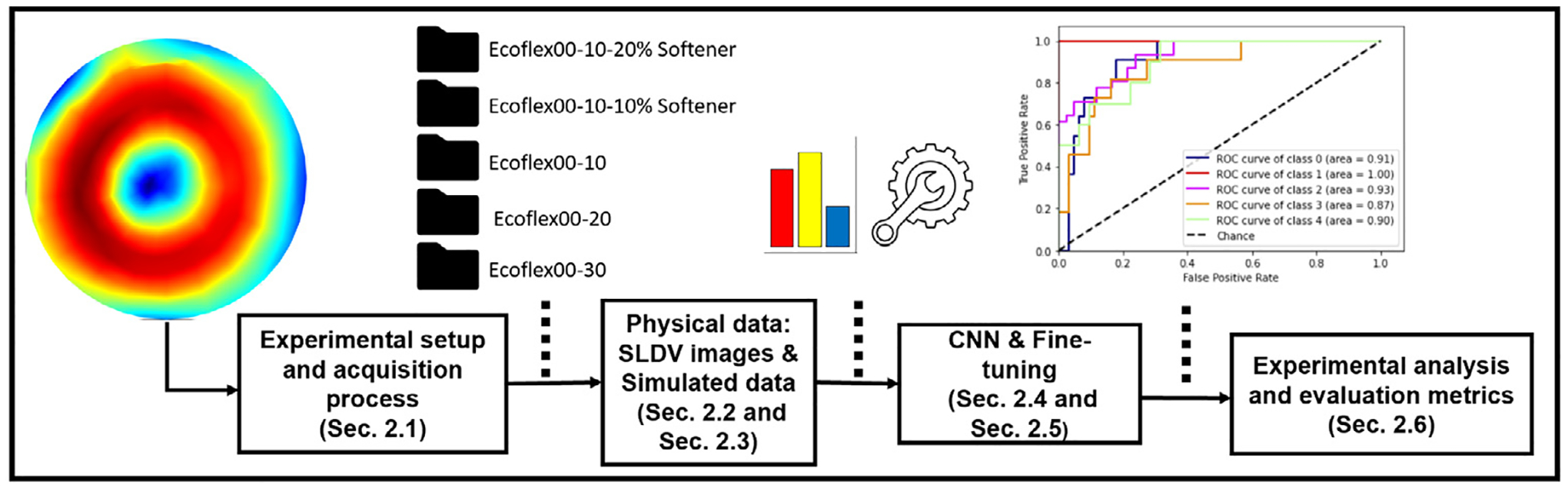
Workflow of the proposed approach for mechanical property classification from Scanning Laser Doppler Vibrometry images with reference to the different steps and relevant sections in which they are described.

This section presents the proposed approach by which the complex shear modulus of different samples was estimated via a CNN (Section 2.4), starting with the data acquisition process using the SLDV (Section 2.1). For each class of data, physical and simulated, two different datasets, in terms of the number of materials to be analyzed and classified, were generated. The first experiment was conducted considering only two classes, in which a class represented a distinct material having a specific shear modulus. In the second experiment, all the five mentioned materials were taken into consideration, having an augmented dataset in terms of the number of classes. This allowed an analysis of more subtle variations of the physical quantity under consideration with respect to the previous experiment. The physical and simulated datasets are described in Sections 2.2 and 2.3, respectively. After pre-training with simulated data, the model was fine-tuned with the physical data, as described in Section 2.5. Evaluation metrics are described in Section 2.6. The workflow of the proposed approach is shown in Fig. 1

### 2.1. Experimental setup and acquisition process

**Fig. 2.**
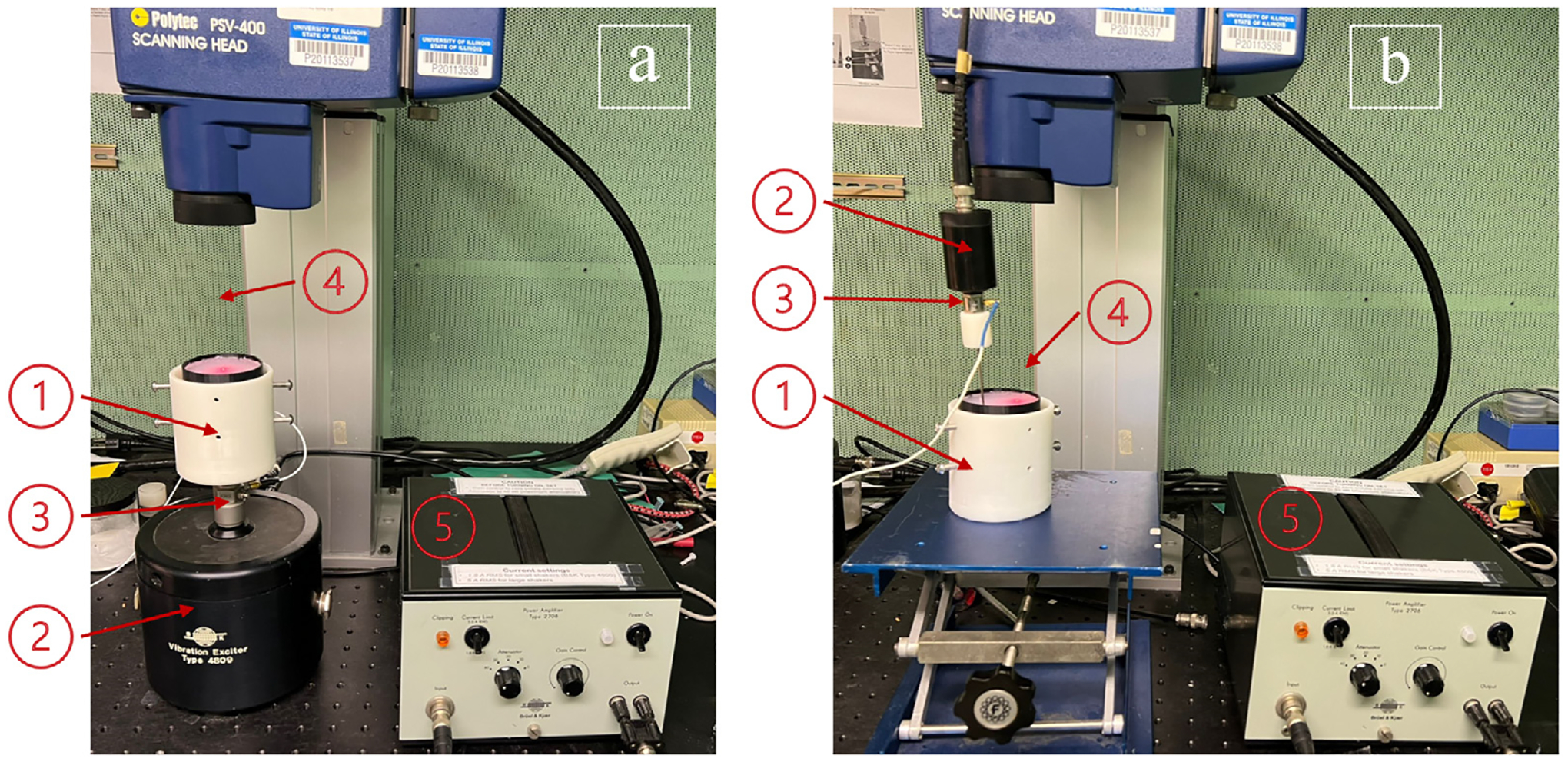
Experimental set-up. (a) **Bench-top shaker** that imparts vertical vibration to the rigid cylinder that surrounds the soft tissue phantom, which otherwise has free boundary conditions on its flat top and bottom surfaces. (b) **Needle shaker** that imparts vertical vibratory motion to a rigid needle inserted vertically into and through the phantom. (1) EcoflexTM phantom. (2) Vibration exciter. (3) Piezoelectric force sensor. (4) LASER, the reflected light is visible on the phantom surface. (5) Amplifier.

The experimental setups used in this work are shown in Fig. 2. The vibration was induced using two different configurations of shakers. First, as indicated in Fig. 2.a, a bench-top shaker (4809, Bruel and Kjaer, Denmark) imparted vertical vibration to the rigid black cylinder that surrounds and contains the soft tissue phantom, which otherwise has free boundary conditions on its flat top and bottom surfaces. This set-up ideally results in an axisymmetric radially converging shear wavefront with respect to the sample. This is actually a Rayleigh–Lamb wavefront given the finite thickness of the phantom, but nearly equals bulk shear wave behavior given the excitation configuration and the phantom thickness versus the driven wavelengths (Brinker et al., 2018; Dore et al., 2023). The other vibration configuration utilized a small shaker (F5B, Wilcoxon, Frederick, MD, USA) that drives a rigid needle that is inserted through the full thickness of the phantom to produce a shear wavefield that radially diverges from the needle and will not be axisymmetric with respect to the phantom unless the needle is oriented along the central axis of the cylindrical phantom. Both shakers were connected to appropriate signal conditioning and received a driving signal from a function generator within the SLDV system (PSV-400, Polytec, Houston, TX, USA) that was synchronized with the SLDV acquisition (Characteristics, 2022).

Ecoflex^™^ (SMOOTH-ON, INC.,USA) (Characteristcs, 2022) was chosen as a phantom material to simulate soft tissue because of its comparable viscoelastic properties and also, importantly, because it is a non-water based polymer with temporally consistent material properties that do not constantly change from one measurement to the next due to water evaporation (Steck et al., 2019). This material also provides a strong and homogeneous adhesion to the container wall, which was the source of vibratory excitation under the axisymmetric configuration described above (Yasar et al., 2013). Another advantage is that, in addition to having different Ecoflex base mixtures with different mechanical properties, the dynamic stiffness can be further fine-tuned by adding a softener to the initial mold liquid. Five phantoms were created – Ecoflex^™^00–10–20% softener, Ecoflex^™^00–10-10% softener, Ecoflex^™^00–10, Ecoflex^™^00–20 and Ecoflex^™^00–30 – that cover a range of stiffness values comparable to biological tissue under healthy and pathological conditions. In this specific study, each phantom had a thickness (height) of 75.0 mm and a diameter of 57.0 mm, and sit within a 3D-printed 1.5 mm thick, nominally rigid, cylinder, but with the flat top and bottom faces of the phantom only in contact with the air.

Cylindrical soft tissue-mimicking specimens of *Ecoflex^™^* were stimulated with 350 Hz sinusoidal vibration during SLDV measurement of motion at an array of points on the flat upper surface, using the two different methods of mechanical vibration excitation (Brinker, 2016). The SLDV uses a helium-neon laser of 632.8 nm wavelength (visible light) and the Doppler effect and interferometry principle, to provide a measure of the surface vertical motion at each measurement point, referenced to the excitation input. Via Fourier-based analysis provided by the SLDV system, the output is a complex-valued velocity for each measurement point at a chosen frequency. This data was exported as a text file and then read into Matlab for additional analysis.

### 2.2. Physical data: SLDV images

Once the acquired data have been processed, each material folder contained the same number of examples. The binary dataset was characterized by the softest and the stiffer material among the five phantoms: Ecoflex^™^00–10-20% softener ad Ecoflex^™^00–30. Afterward, the model was trained to solve a multi-class classification problem, where the experimental dataset was composed of all the five phantoms.

Images were cropped to exclude image boundaries and avoid any irregularity and numerical instability (Rivera and Andrade, 2000; Carcione, 1994). Only 13 images for each class of material were available, which is not enough to compose a useful and reliable data set for a future classification task. The limitation of having a small amount of data is in part due to the viscoelastic property of silicon materials, which means that it essentially has different material properties at different frequencies. Therefore, a patch extraction procedure was performed on each image to dramatically increase the dataset size without causing a loss of information: indeed each patch sector still contained a significant amount of information to be a discriminant example inside its specific class of material. After this pre-processing step, the dataset comprised 52 samples with a dimension of 71 × 11 × 2, where the first channel refers to the real part and the second channel to the imaginary part of the temporal harmonic of the wavefield data.

**Fig. 3.**
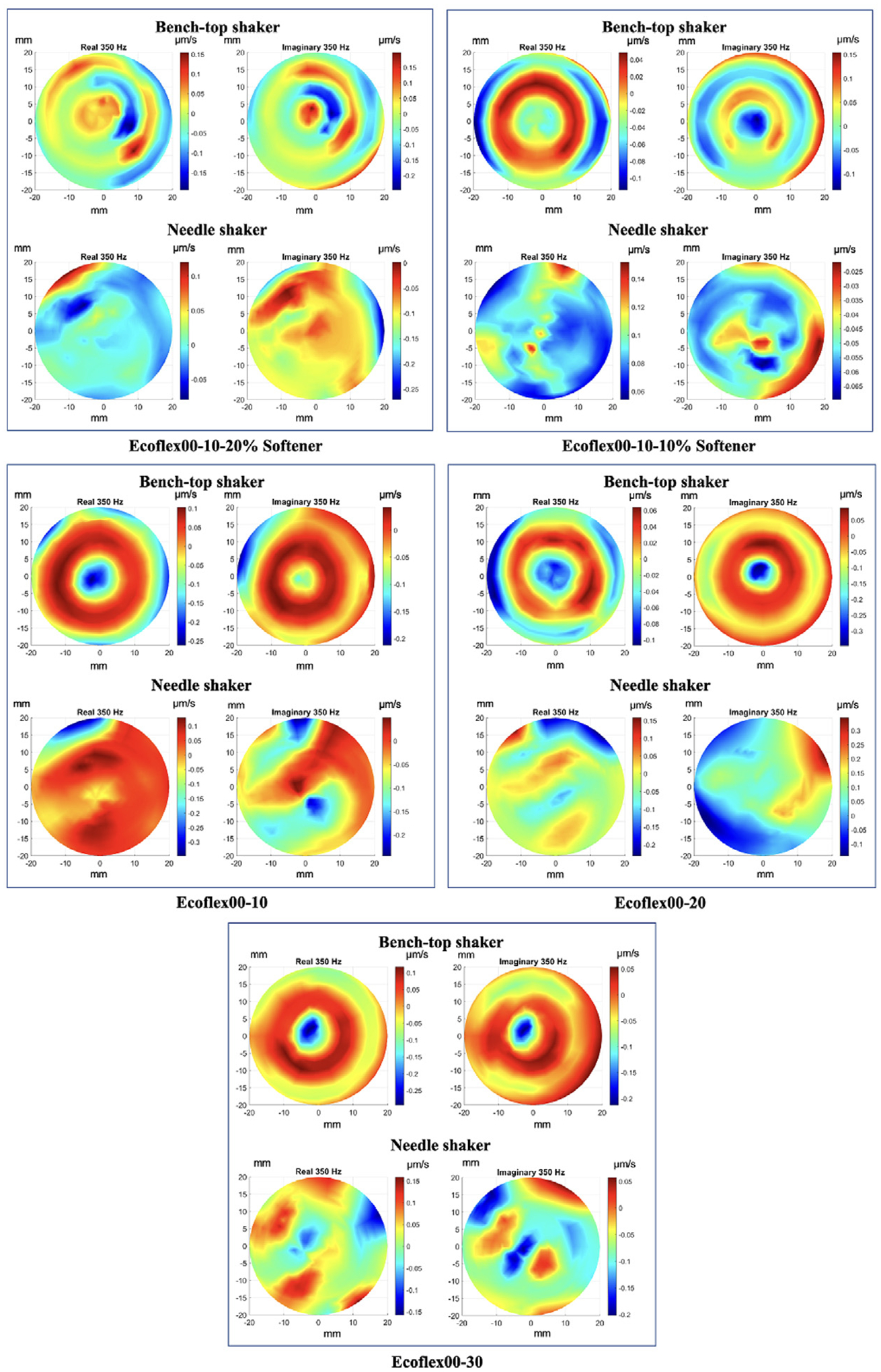
Examples of interpolated maps of surface normal motion at an instant in time from physical samples belonging to Ecoflex^™^00–10-20% softener, Ecoflex^™^00–10-10% softener, Ecoflex^™^00–10, Ecoflex^™^00–20, and Ecoflex^™^00–30 class. The “imaginary” images are 90 degrees out of phase from the “real” images.

The following images (Fig. 3) show some examples of the interpolated map of snapshots of surface normal motion considering the five different materials. The images of the imaginary part are 90 degrees out of phase from the images of the real part of the wavefield. We expected to obtain different images according to the shaker utilized for the mechanical vibration; however, we were aware that the shear modulus of the material would not change based on the used shaker because the external excitation does not change the intrinsic mechanical properties of the material. The use of two shakers simply increased the number and variation of available images within the dataset.

The splitting of the data set was performed considering the three subsets: training, validation, and test set. Among the entire data set, the ratio between the effective training and test set was 80:20. A portion of the training set was used for the validation set in a ratio of 70:30. Offline data augmentation on the training set was applied considering transformations as: reflecting, cropping, translating, and changing the brightness of the image. All the transformations applied in this study are related to the class of affine transformations of the original image, where the safety of these manipulations refers to its likelihood of preserving the label post-transformation (Perez and Wang, 2017).

### 2.3. Simulated data

As mentioned in Section 1.1, due to the limited amount of physical data and considering that data availability is a crucial element in the DL field, the model was pre-trained using synthetic data. Indeed, DL models improve their performance according to the number of samples available for learning (Roh et al., 2019). Only 52 physical samples for each class of material were obtained and to cope with this lack of data and achieve consistency for the entire research process, simulated data were generated with the same size and frequency characteristics as the physical one. The hypotheses assumed to generate this mechanical wavefield and to obtain an analytical solvable approximation of Green’s function for the wave equation were homogeneity and isotropy (Royston, 2021).

The first set of simulated data is characterized by two classes of shear storage modulus, 1 kPa, and 5 kPa, therefore the model was trained to solve a binary classification problem. The ratio of shear loss to shear storage moduli fixed at 0.15. The shear moduli were chosen to cover a range of values similar to normal and pathological soft tissues. The multi-class classification considers a broader range of stiffness, and the orthogonal shear elasticity *μ* of each example was chosen randomly from a uniform distribution within a range of 1 to 5 kPa considering only integer numbers (Murphy et al., 2018), with a ratio of shear loss to shear storage moduli fixed at 0.15.

As the simulated representations of physical data were used to train the model, a simulation of mutable source locations was implemented as well. The source location has been computed to vary from each reconstructed sample, where a single point source was placed in a random location within a circular shell at a variable distance from the center of the patch. In our specific case, the propagation of the wave is a function of the complex shear modulus and the source location, where changing the source location of the mechanical vibration changes the structure of the wavefield. However, the estimated shear modulus remains the same.

All the simulation samples were created using functions implemented in MATLAB (Mathworks, Natick, MA, USA).

### 2.4. Convolutional neural network

**Fig. 4.**
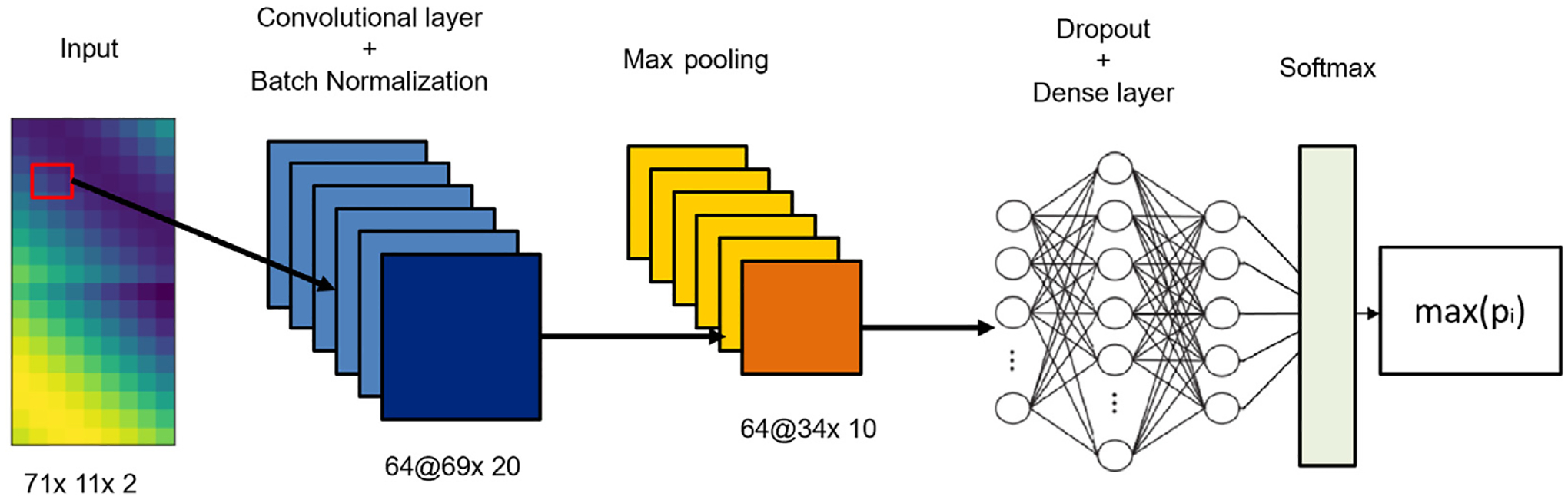
Graphic illustration of the CNN architecture adopted in this research. Feature maps number and dimensions are reported. The estimated class at the output will be the one with the highest value of probability (*p*_*i*_) associated.

The model architecture design consists of a simple block containing the sequence of a convolutional, a batch normalization, and a max-pooling layer, as illustrated in the following Fig. 4. The dimension of the filter that performs an operation of convolution with the input map in the convolutional layer is 3 × 3 with a stride = 1 and no padding. At each feature map, a rectified linear unit (ReLU) was applied, followed by a batch normalization, and by the operation of max pooling with kernel = 2 and stride = 2. The learned features are the input of a fully connected neural network composed of two dense layers of 1024 and 512 neurons, alternated by a dropout layer with a dropout rate set equal to 0.5. The last layer is the Softmax which has been applied both for binary and multi-class classification tasks. The softmax function is a combination of multiple sigmoid functions (Sharma et al., 2017), and for every data point of all the individual classes it returns a probability value. In this situation, the output layer of the network will have the same number of neurons as the number of classes in the target (Martins and Astudillo, 2016).

The implementation of this CNN model was performed through the access to the computing resources of Colab, including GPUs. The CNN model was built and trained using Keras with a TensorFlow backend (Chollet, 2017).

### 2.5. Fine tuning

After having allowed the transfer of weights from the pre-trained network on simulated data to a new network, the 2D convolutional layer and the max-pooling layer of the model were frozen, while the fully connected layer was trained at a lower learning rate on physical data. Moreover, the softmax classifier was removed and substituted with a new one to begin the fine-tuning procedure. The new softmax layer was trained from scratch with the samples from the experimental dataset in order to ensure a reliable classification without any bias from the simulated one (Reyes et al., 2015). In the last few layers, the learning rate was set to decrease with the epochs from 0.001 to 0.0001 and the batch size was set to 12 (Zhou et al., 2017).

### 2.6. Experimental analysis and evaluation metrics

The performance of the proposed CNN was first computed in two different experiments of simulated and physical data with increasing complexity. The first two experiments aimed to verify the ability of the model to correctly classify physical samples acquired using the SLDV, firstly considering two different materials (*Ecoflex^™^*00–10-20% softener and *Ecoflex^™^*00–30), labeled as 0. and 1., respectively, and afterwards, with all the five different materials (*Ecoflex^™^*00–10-20% softener, *Ecoflex^™^*00–10-20% softener, *Ecoflex^™^*00–10, *Ecoflex^™^*00–20 and *Ecoflex^™^*00–30), labeled as 0., 1., 2., 3. and 4. respectively. For the first experiment, a binary cross-entropy function, a learning rate equal to 0.0001, and a batch size of 10 were used, while for the second experiment categorical cross-entropy as loss function, 0.001 as learning rate, and a batch size equal to 16 were selected. For both experiments, the number of epochs was set to 30 and Adam as optimizer was used. The results of the CNN predictions with and without applying the transfer learning and fine-tuning techniques, described in Section 2.5, were compared.

The ability of the implemented CNN architecture to recognize and estimate the shear modulus of the tissue-mimicking materials was verified through some parameters of the model’s performance. Specifically, the indexes considered to evaluate the performance of the model were accuracy, recall, precision, and the F1-score. For completeness, the Area Under the Curve (AUC) of the Receiver Operating Characteristic (ROC) curve was provided for each assessment.

(1) $$\text{Accuracy}=\frac{1}{n}\sum_{i=1}^{n}\frac{TP_{i}+TN_{i}}{TP_{i}+TN_{i}+FP_{i}+FN_{i}}$$

(2) $${\text{Recall}}_{i}=\frac{TP_{i}}{TP_{i}+FN_{i}}$$

(3) $${\text{Precision}}_{i}=\frac{TP_{i}}{TP_{i}+FP_{i}}$$

F1-score is computed as the weighted average of the precision and recall. In this work, since we are dealing with a multi-label problem, we take into consideration the macro-averaged F1-score defined as:

(4) $$F1-\text{score}=\frac{1}{n}\sum_{i=1}^{n}\frac{2*{\text{Precision}}_{i}*{\text{Recall}}_{i}}{{\text{Precision}}_{i}+{\text{Recall}}_{i}}$$

where *TP* stands for true positive, (i.e. the predicted class was correct), true negative (*TN*) (i.e. correctly classified negative cases), false positive (*FP*), and false negative (*FN*) (i.e. the incorrectly classified negative cases and the incorrectly classified positive cases, respectively).

## 3. Results

In the following section, some examples of the simulated data (Section 3.1) are presented. Then, the classification results obtained from training and validating the CNN architecture to the datasets are described considering the two experiments (Sections 3.2 and 3.3). By reviewing the learning curves of the model, a first overview of the learning procedure was provided concerning, for instance, overfitting and whether the training and validation datasets are suitably representative.

### 3.1. SLDV images and simulated data

**Fig. 5.**
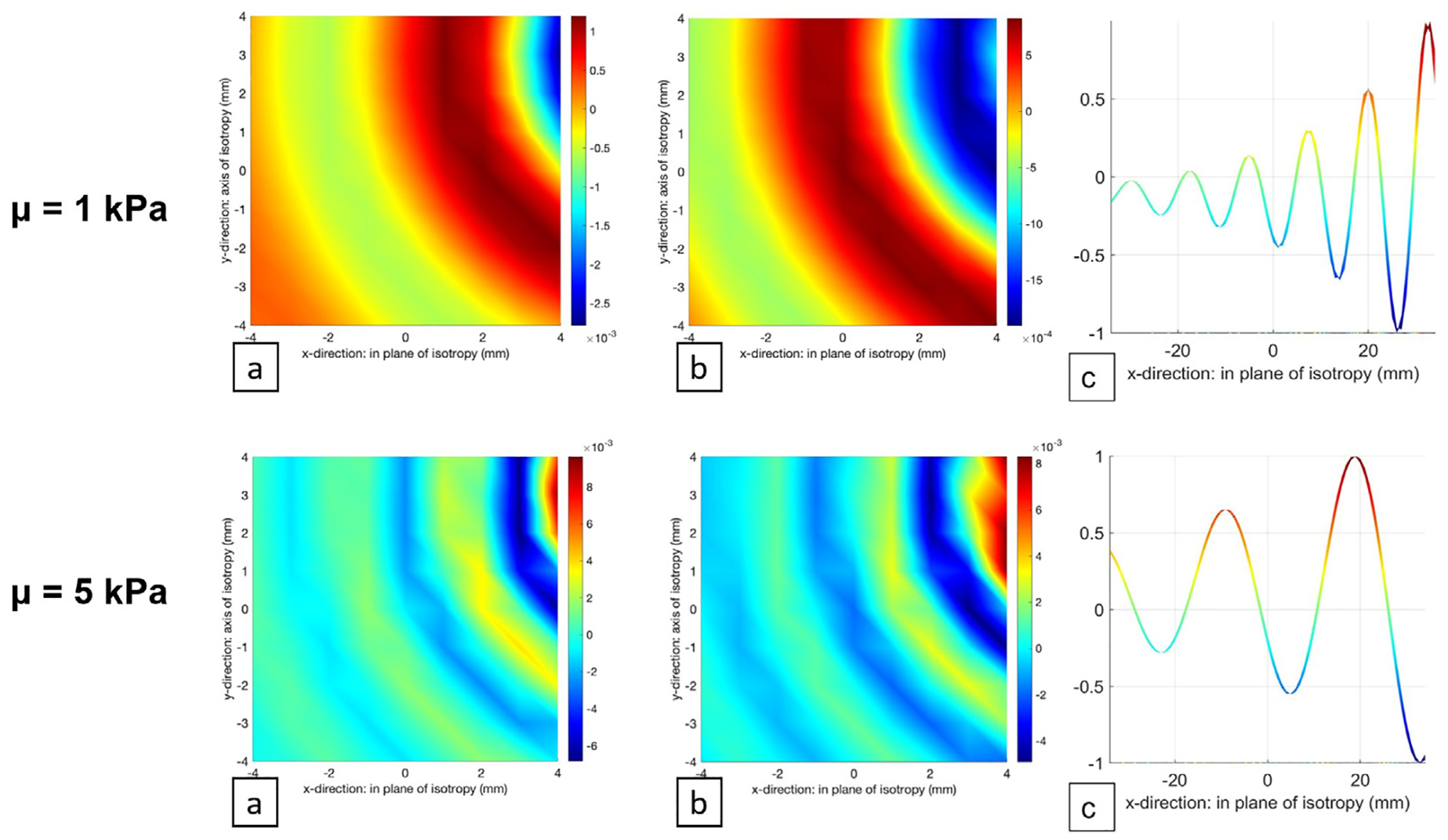
Examples of simulated samples for class *μ* = 1 kPa and class *μ* = 5 kPa materials, and the corresponding wavelength profile. For each simulated sample: (a) real part, (b) imaginary part, and (c) wavelength profile.

The images in Fig. 5 are different snapshots of the real and the imaginary part of the simulated wavefield for class *μ* = 1 kPa and class *μ* = 5 kPa (ratio of shear loss to shear storage moduli fixed at 0.15), where the distances between two consecutive wavefronts are evident and representative of each class. In addition, a profile of the wavelength is provided, where the correlation between the wavelength and the shear modulus of the specific materials can be observed. The wavefield has been displayed as a sinusoidal plane wave. The color bar shows the values of velocity measured in μm/s in the specific instant in which the snapshot has been taken, considering the entire wave propagation period.

### 3.2. First experiment

**Fig. 6.**
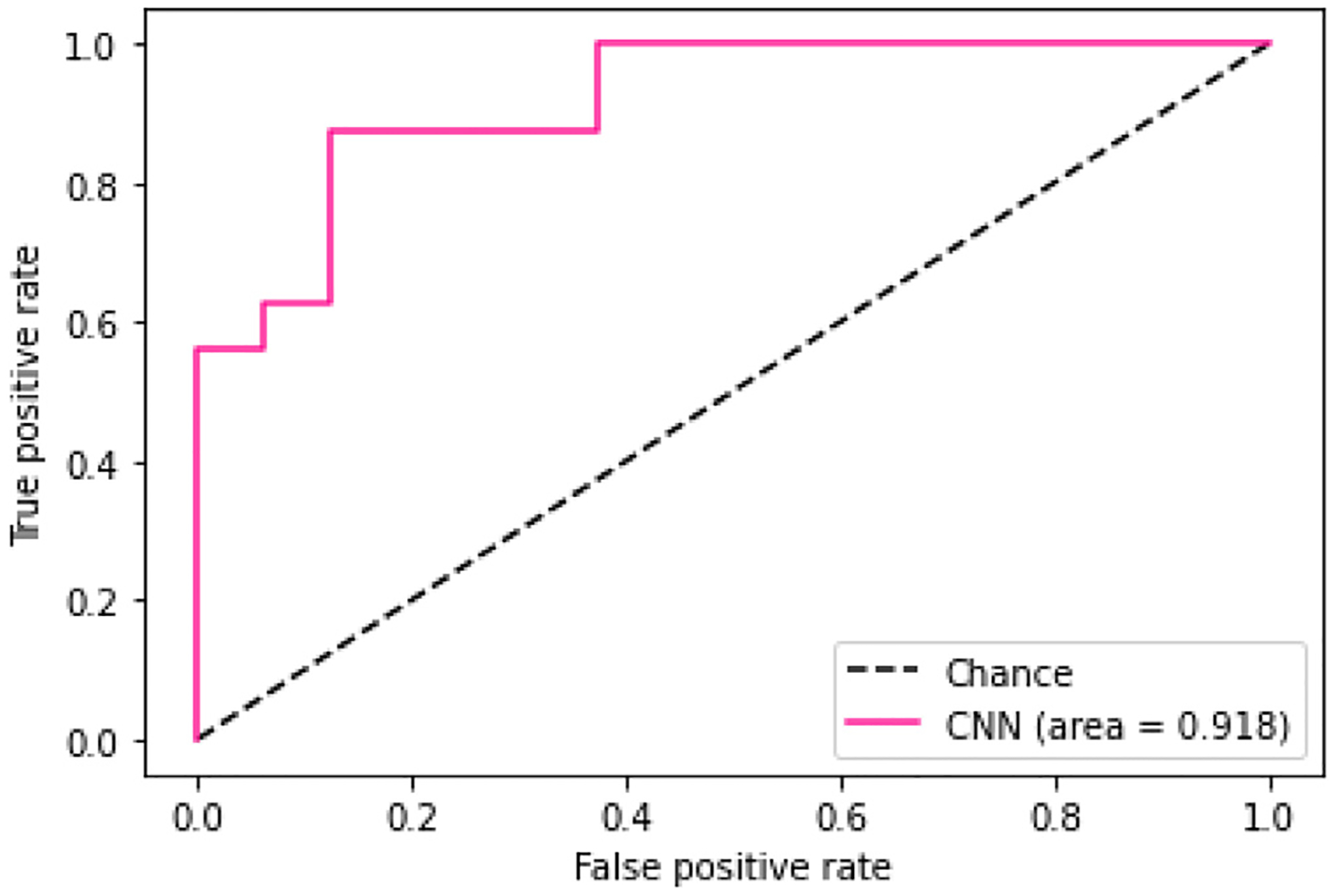
The Receiver Operator Characteristic (ROC) curve evaluates the binary classification model.

The first evaluation aimed to verify the ability to correctly classify physical samples acquired using the SLDV from two different materials, Ecoflex^™^00–10-20% softener and Ecoflex^™^00–30. After applying the fine-tuning, i.e., pre-training the model with the synthetic data, the loss and accuracy curves of the training and validation sets showed a good fit over the number of epochs. For this experiment, precision, recall, f1-score, and accuracy of the resulting binary classification performance were 0.85, 0.84, 0.84, and 0.84, respectively. The predictive power of the implemented model was analyzed and the value of the normalized AUC of the ROC curve was 0.918 (Fig. 6).

A visual comparison of the macro-averaged values of accuracy, precision, recall, and f1-score is shown in the table (Table 1), comparing the achieved results using the CNN without and with having applied the fine-tuning.

### 3.3. Second experiment

**Fig. 7.**
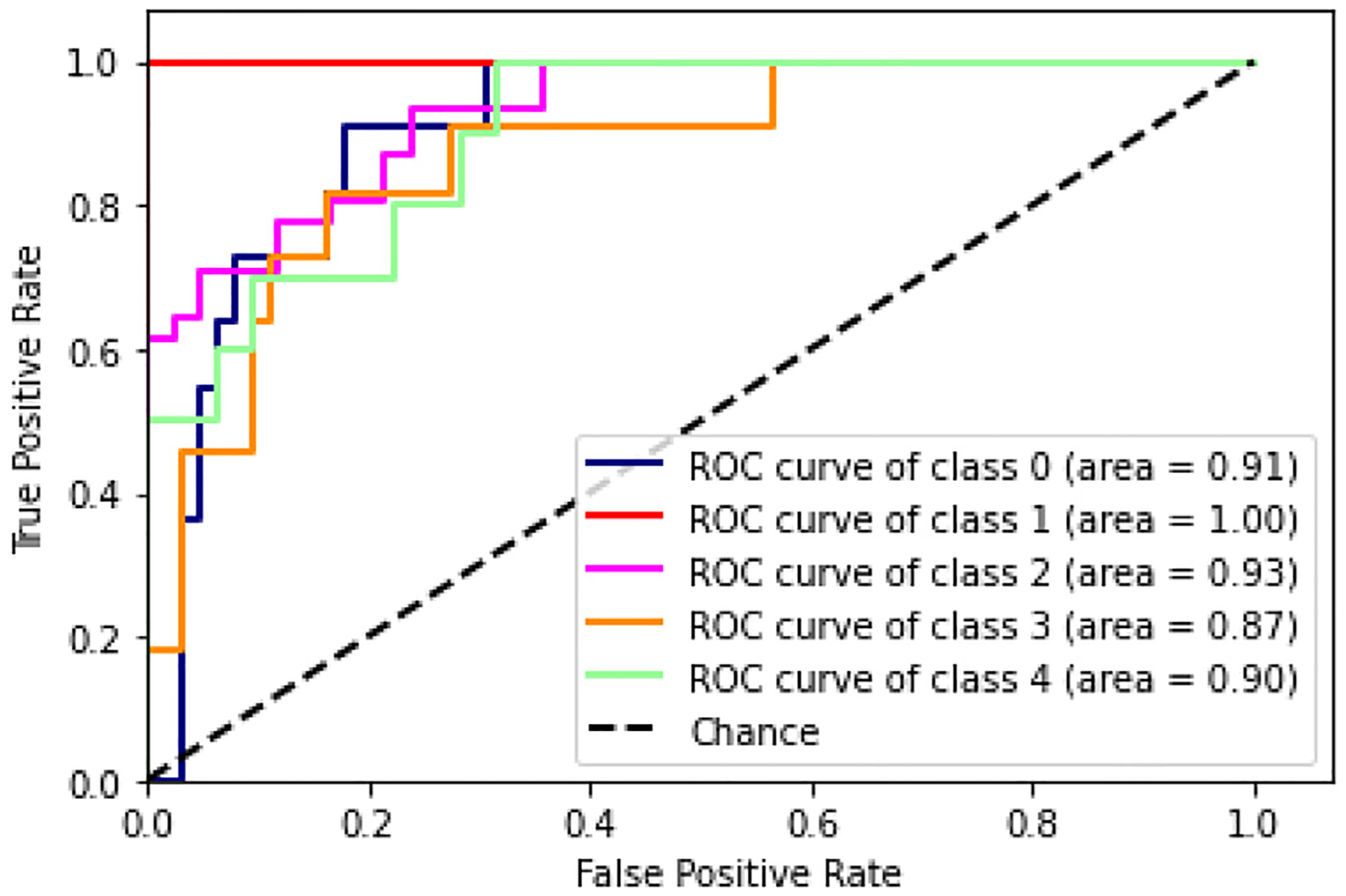
The Receiver Operator Characteristic (ROC) curve evaluates the implemented CNN in a “one-vs-rest” mode, i.e. each class of physical data against all the others at the same time. The rate between true positives against false positives at various threshold values is represented for each assessment. The Area Under the Curve (AUC) measures the ability of the model to distinguish a specific class with respect to the others.

Even when increasing the complexity of the problem, and intro-ducing three additional classes with respect to the previous setting, the training and validation loss curves still show a good fit. Both curves reach a point of stability over the time domain after training for 30 epochs. As in the previous setting, precision, recall, f1-score and accuracy of the resulting 5-class classification were computed. The obtained results were 0.76, 0.72, 0.72 and 0.77, respectively. In addition, the associated ROC curve is reported in Fig. 7 where the computed normalized AUC macro-average across the five classes was 0.92.

The macro-averaged values of the evaluation metrics are summarized in the following table (Table 2), highlighting the differences in having or not applied the fine-tuning technique.

## 4. Discussion

Pre-training the CNN using simulated data and fine-tuning the model utilizing physical data led to well-classified realistic values for the wave speed traveling on the surface of these silicon materials. As expected, the binary classification achieved better results than the multi-class classification. However, the CNN architecture was still able to distinguish among the different classes, showing a significant rate of well-predicted data concerning both binary classification and the multi-class classification. The binary classification could resemble the healthy and pathological tissue, while the multi-class classification could simulate several stages of pathology progression. In this study, the classification criteria are the pixel values of each image since the spatial distribution of these values will depend on shear wave speed and attenuation, which in turn depend on the shear storage and loss moduli at the frequency being used. Variations in the complex shear modulus produce variations in the shear wave speed and attenuation across the surface of the material. In addition to this, the estimation of the correct material’s shear modulus was more challenging in the context of the 5-class classification due to the more subtle and significant variations between one class and the next one. These results were also supported by the AUC values extracted from the ROC curves in Fig. 7, where the multi-class model was evaluated in a “One vs. Rest” mode. In this scenario, the results can help to understand which classes the model is struggling to describe. The model presented a good ability to distinguish all the classes except for class 3 which is represented by the material *Ecoflex^™^*00–20. Indeed the rate between true positive against false positive of the *Ecoflex^™^*00–20 against all the other materials was 0.87 compared to the other AUC values that were 0.91 (*Ecoflex^™^*00–10-20%), 1.00 (*Ecoflex^™^*00–10-10%), 0.93 (*Ecoflex^™^*00–10) and 0.90 (*Ecoflex^™^*00–30).

Even if the implemented CNN achieved good performance in all two settings, however, some limitations of this study will serve as the subject of future works and investigation. Indeed, the five considered materials were all homogenous and isotropic. This resulted in the generation of less realistic and diverse examples, which are fundamental for training deep networks in the field of medical images. Applying this classification algorithm to materials that present anisotropic characteristics would likely generate a more complex dataset. Accordingly, two elastography-based studies accounted for more realistic materials, both in-vitro and in-vivo, to be as close as possible to human soft tissues. Hou et al. used chicken breast muscle tissue as the material of interest to account for anisotropy, which is an intrinsic property of striated muscle, to estimate the mechanical properties of MRE images via a CNN. Zhou et al. (2017). Guidetti et al. (2019) simulated the skeleton muscle using silicon phantoms with an embedded fiber structure.

Other considerations could be made about the generation of materials that emulate better the behavior of different soft tissues; indeed the samples were acquired without applying any static strain to the materials, which is also known to affect wave behavior (acoustoelastic effect) (Salehabadi et al., 2023; Crutison et al., 2022).

Furthermore, the implementation of more complex functions to generate the simulated data may improve the performance of the classification model. Even Murphy et al. observed that the use of more advanced simulations and more realistic MRE-like noise may further improve accuracy in vivo and allow for NNI estimation of additional mechanical parameters (e.g., storage and loss moduli or damping ratio) (Murphy et al., 2018).

## 5. Conclusion

Classification of the shear modulus of different silicon materials using a convolution neural network was evaluated using experimental data acquired via scanning laser Doppler vibrometry, a dynamic elastography technique. Preliminary results showed that the implemented CNN model was capable of distinguishing and predicting five different phantom materials that could resemble intermediate stages of a pathology, where the discriminant features are the changes in the wave motion on the tissue surface and the correlated shear modulus. In conclusion, this study demonstrated the reliability of the CNN for the estimation of mechanical properties from images acquired through a non-contact, non-invasive dynamic elastography technique, potentially improving upon the use of traditional inversion algorithms.

## Figures and Tables

**Table 1:** Comparison of the performances of two different models trained on the same 2-class of physical data without and with fine-tuning process.

Model	Accuracy	Precision	Recall	F1-score
CNN (no fine-tuning)	0.576	0.590	0.580	0.580
CNN (fine-tuning)	0.844	0.850	0.840	0.840

**Table 2:** Comparison of the performances of two different models trained on the same 2-class of physical data without and with fine-tuning process.

Model	Accuracy	Precision	Recall	F1-score
CNN (no fine-tuning)	0.547	0.560	0.510	0.500
CNN (fine-tuning)	0.767	0.760	0.720	0.720

## Data Availability

Data will be made available on request.
